# Is Praecox Feeling at risk of extinction?

**DOI:** 10.1192/j.eurpsy.2022.569

**Published:** 2022-09-01

**Authors:** A. Duarte, C. Laginhas, A. Lourenço, I. Simões, P. Martins

**Affiliations:** 1 Centro Hospitalar Lisboa Norte, Psychiatry, Lisboa, Portugal; 2 Hospital Egas Moniz, Psychiatry, Lisbon, Portugal

**Keywords:** schizophrénia, Psychopathology, Praecox Feeling, diagnostic judgment

## Abstract

**Introduction:**

Praecox feeling (PF) is a characteristic feeling of bizarreness or unease that a psychiatrist experiences when facing a patient with schizophrenia. This term, proposed by Rumke in 1941, was considered an important feature of a schizophrenia diagnosis. However, since the movement toward operational diagnostic methods in the late 1970s, it has fallen out of use.

**Objectives:**

This work aims to discuss the role of Praecox Feeling in the clinical approach to schizophrenia diagnosis.

**Methods:**

PubMed database was searched using combinations of the terms “praecox”, combined with “feeling” and “schizophrenia”.

**Results:**

PF is sometimes experienced silently before the patient participates verbally. An experienced and attentive clinician can intuitively feels changes in the body posture, facial expression, the tone of the voice, motor behavior, and attitude that could look insignificant, but as a whole they present the patient as “definitely un-understandable.” Although there is lacking evidence to sustain the rehabilitation of the PF as a reliable and valid clinical criterion consistent with the operational approach, a broader scientific approach is called for. PF should not be trivialized, as is sometimes the case, into a quick diagnosis but could be a real determinant of medical decision.

**Conclusions:**

Even though there may not be sufficient evidence to consider it valid clinical diagnostic criteria, it still appears to play an important role in the clinical decision-making process and should not be underestimated or stigmatized. This concept is not completely subjective and does rely on objective information, such as the patient’s behaviour and body language.

**Disclosure:**

No significant relationships.

